# A New Mouse Model to Explore Therapies for Preeclampsia

**DOI:** 10.1371/journal.pone.0013663

**Published:** 2010-10-27

**Authors:** Abdulwahab Ahmed, Jameel Singh, Ysodra Khan, Surya V. Seshan, Guillermina Girardi

**Affiliations:** 1 Department of Biology, York College, City University of New York, New York, New York, United States of America; 2 Department of Pathology, Weill Cornell Medical College, New York, New York, United States of America; Institut Jacques Monod, France

## Abstract

**Background:**

Pre-eclampsia, a pregnancy-specific multisystemic disorder is a leading cause of maternal and perinatal mortality and morbidity. This syndrome has been known to medical science since ancient times. However, despite considerable research, the cause/s of preeclampsia remain unclear, and there is no effective treatment. Development of an animal model that recapitulates this complex pregnancy-related disorder may help to expand our understanding and may hold great potential for the design and implementation of effective treatment.

**Methodology/Principal Findings:**

Here we show that the CBA/J x DBA/2 mouse model of recurrent miscarriage is also a model of immunologically-mediated preeclampsia (PE). DBA/J mated CBA/J females spontaneously develop many features of human PE (primigravidity, albuminuria, endotheliosis, increased sensitivity to angiotensin II and increased plasma leptin levels) that correlates with bad pregnancy outcomes. We previously reported that antagonism of vascular endothelial growth factor (VEGF) signaling by soluble VEGF receptor 1 (sFlt-1) is involved in placental and fetal injury in CBA/J x DBA/2 mice. Using this animal model that recapitulates many of the features of preeclampsia in women, we found that pravastatin restores angiogenic balance, ameliorates glomerular injury, diminishes hypersensitivity to angiotensin II and protects pregnancies.

**Conclusions/Significance:**

We described a new mouse model of PE, were the relevant key features of human preeclampsia develop spontaneously. The CBA/J x DBA/2 model, that recapitulates this complex disorder, helped us identify pravastatin as a candidate therapy to prevent preeclampsia and its related complications. We recognize that these studies were conducted in mice and that clinical trials are needed to confirm its application to humans.

## Introduction

Preeclampsia (PE) is a pregnancy-specific, multisystemic disorder that occurs in about 1 in 12 of all live-birth pregnancies in the United States, and it is a leading cause of maternal and fetal mortality and morbidity [1], [2]. More than 200,000 American women per year develop PE (a number equal to the number of women affected by breast cancer). It is the most common reasons for a woman to die during pregnancy. This syndrome has been known to medical science since ancient times. However, despite considerable research, the cause/s of PE remain/s unclear, and there is no effective treatment. Development of an animal model that recapitulates this complex pregnancy-related disorder may help to expand our understanding and may hold great potential for the design and implementation of effective treatment.

DBA/2-mated female CBA/J mice (CBA/J × DBA/2) are a well-studied model of immunologically mediated pregnancy loss [3], [4].We previously described the important contribution of complement activation to adverse pregnancy outcomes in this model [5]. In these abortion-prone matings, generation of the anaphylotoxin C5a and increased tissue factor expression, causes dysregulation of angiogenic factors and abnormal placental development [5], [6]. Diminished giant trophoblast cells, diminished placental perfusion and bad pregnancy outcomes were observed in CBA/J x DBA/2 mice [5], [6]. Knowing that defective placentation due to increased antiangiogenic soluble receptor for vascular endothelial growth factor 1 (sFlt-1) can trigger PE in rodents and women [7], [8] and that inflammation has been implicated in the pathogenesis of PE [9], [10] led as to investigate if the CBA/J x DBA/2 mating model constitutes a model of PE. Here we show that the CBA/J x DBA/2 model of recurrent miscarriage is also a model of PE that shares many features with human PE. With the use of this unique mouse model that spontaneously develops the pathological changes associated with PE, we examined the beneficial effects of pravastatin in preventing the onset of the characteristic features of PE. Pravastatin restored angiogenic balance and prevented the onset of the key preeclamptic symptoms in CBA/J x DBA/2 mice.

## Results

### Bad pregnancy outcomes in first pregnancy

We previously reported that embryos derived from mating CBA/J females with DBA/2 males showed an increased frequency of resorption when compared to control matings BALB/c-mated CBA/J female mice and that surviving fetuses from CBA/J x DBA/2 matings showed consistent and significant intrauterine growth restriction (IUGR) [5]. The expressivity of the phenotype (fetal loss and IUGR) in CBA/J x DBA/2 matings was constant. 100% DBA/2-mated CBA/J mice presented increased fetal resorption frequency and smaller fetuses.

**Figure 1:**
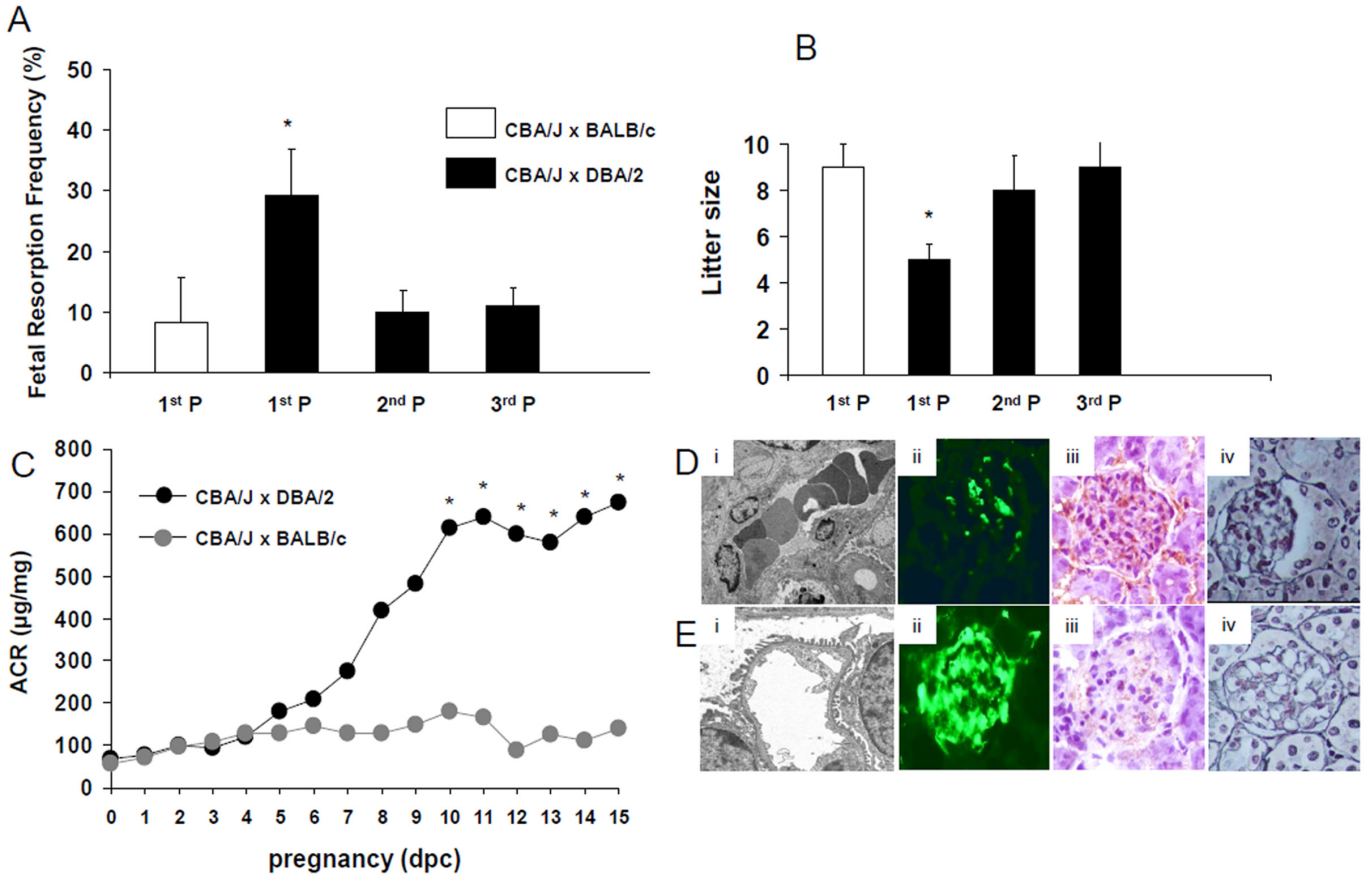
Preeclamptic features in CBA/J x DBA/2 matings. Increased fetal resorption frequency (A) and decreased litter size (B)) were observed only in first pregnancies in DBA/2 mated CBA/J mice. Normal pregnancies (not different from control matings CBA/J x BALB/c) were observed in second (2^nd^ P) and third (3^rd^ P) pregnancies with the same male. Data are mean values plus or minus SD. (C) In CBA/J x DBA/2 mice, a time-course increase in urine albumin to creatinine ratio (ACR), that reaches statistic significance at day 10 of pregnancy, was observed (*p<0.05 vs CBA/J x DBA/2). Albuminuria was not observed in control matings CBA/J x BALB/c mice. N = 6-8 mice/experimental group (Di) Transmission electron micrographs of glomeruli from DBA/J mated CBA/J mice (original magnification 10,000x) show swelling of glomerular endothelial cells and loss of fenestrations. Numerous red blood cells trapped between swollen endothelial cells were observed occluding the glomerular capillaries. No signs of endothelial injury, well preserved fenestrations and widely patent glomerular capillary lumina are observed in kidneys from CBA/J x BALB/c mice (control group) (Ei). (Dii) Kidney perfusion studies reveal diminished blood perfusion (the fluorescent tracer did not accumulate in the glomerular capillaries) in glomeruli from CBA/J x DBA/2 mice with abnormal pregnancies compared to control CBA/J x BALB/c matings (Eii). (Diii) Increased fibrin staining in glomeruli from CBA/J x DBA/2 mice when compared to CBA/J x BALB/c mice (Eiii). (Div and Eiv) Jones methenamine silver staining. Diffusely and irregularly thickened glomerular basement membranes were observed in some glomeruli in CBA/J x DBA/2 mice (20%) (Fig 1Div) compared to BALB/c mated CBA/2 females (Fig 1Eiv). Kidneys from 5-6 mice were studies in each experimental group.

PE is twice as common in primigravid women as in women having second or later pregnancies, suggesting an immune cause [11]. Despite the increased fetal resorption rate observed in first mating of CBA/J females with DBA/2 males, increased fetal death and growth restriction was not observed in the second and third pregnancies (**Fig 1A**). In addition, IUGR was not observed in the 2^nd^ and 3^rd^ pregnancies in CBA/J x DBA/2 mice. Fetal weights in second and third pregnancies (356±36 mg and 370±45mg respectively) were not different from those observed in control mating pairs CBA/J x BALB/c (348±41 mg) (n = 140-160 fetuses/experimental group). Fetal weight in CBA/J x BALB/c matings did not change in relation to the number pregnancies (data not shown) (n = 120 fetuses/group). A group of mice was studied until birth and litter sizes were recorded (**Fig 1B**). In CBA/J x DBA/2 mice the litter sizes in the first pregnancy were smaller compared to the 2^nd^ and 3^rd^ pregnancy. Litter sizes in 2^nd^ and 3^rd^ pregnancy were not different from values observed in control CBA/J x BALB/c matings (**Fig 1B**). 6 to 8 mice were studied in each experimental group.

### Increased albuminuria and glomerular endotheliosis in CBA/J x DBA/2 mice

**Figure 2:**
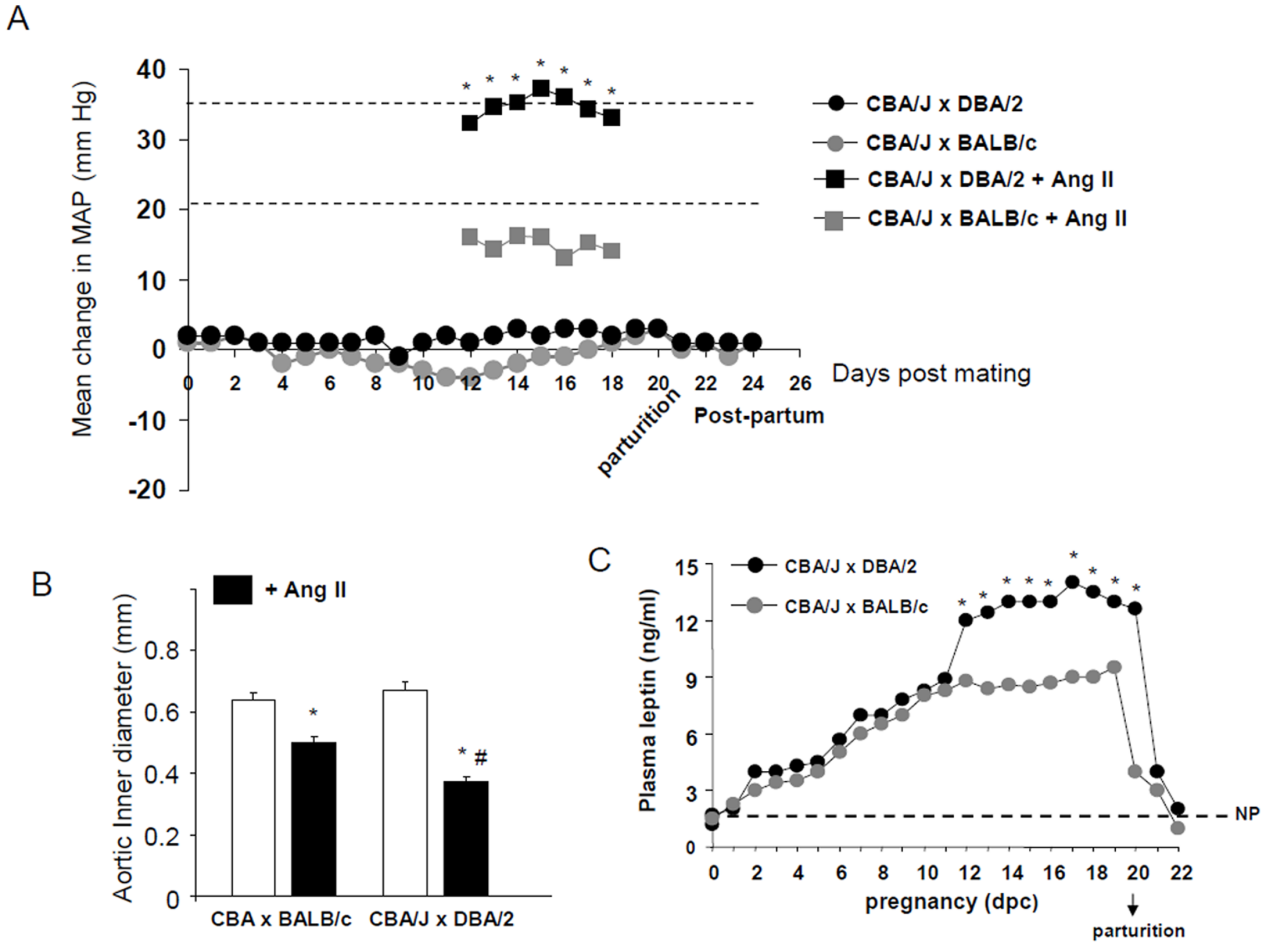
MAP and contractile response to angiotensin II in CBA/J x DBA/2 mice. (A) No significant increase in MAP was observed in CBA/J x DBA/2 compared to CBA/J x BALB/c control matings. Data were expressed as change in MAP from mating day. CBA/J x DBA/2 mice showed an increase response to an acute a bolus injection of AngII compared to CBA/J x BALB/c mice (*p<0.01). The response to Ang II was expressed as the mean change in MAP from basal values before Ang II injection. (B) Aortic rings from CBA/J x DBA/2 and CBA/J x BALB/c contracted in response to Ang II (* p<0.05). Aortic rings from CBA/J x DBA/2 mice showed increased reduction of the inner diameter in response to Ang II (# different from CBA/J x BALB/c. P<0.05). N = 8–10 mice/experimental group. (C) Staring at day 11 of pregnancy, plasma leptin levels were increased in CBA/J x DBA/2 mice when compared to control CBA/J x BALB/c matings (* p<0.01). This increase was maintained until delivery. Leptin levels in CBA/J x DBA/2 and control matings drop to pre-pregnancy values after parturition. N = 6–8 mice/experimental group.

The presence of proteinuria is used to confirm the diagnosis of PE [12]. In CBA/J x DBA/2 mice, a time-course increase in urine albumin to creatinine ratio (ACR), that reaches statistic significance at day 10 of pregnancy, was observed (**Fig 1C**). On day 15 of pregnancy ACR in CBA/J x DBA/2 mice was 5 times higher than that measured in control mice (Figure 2A). In CBA/J x BALB/c mice a very slight increase in ACR was observed through pregnancy (**Fig 1C**). Proteinuria was not observed in second and third pregnancies in CBA/J x DBA/2 mice (data not shown).

Endotheliosis, an inflammation of the glomerular endothelium, is a frequent renal lesion observed in women with PE [12]. Electron microscopic analysis (EM) was performed to identify endothelial injury in CBA/J x DBA/2 mice. EM examination of renal glomerular capillaries in CBA/J xDBA/2 mice showed endothelial swelling with reduction of endothelial fenestrations (**Fig 1Di**). Swollen glomerular endothelial cells occupied a wide area of the capillaries lumina and clumping of red cells occluded the capillary lumina in 30% of the glomeruli in CBA/J x DBA/2 mice. In the control CBA/J x BALB/c mating, glomeruli presented open capillary lumens and intact endothelial cells (**Fig 1Ei**). Women with PE have a decrease in glomerular filtration and renal blood flow [13]. Decreased renal blood flow was observed in some kidneys from CBA/J x DBA/2 mice (30%) at day 15 of pregnancy (**Fig 1Dii**) when compared to CBA/J x BALB/c mice (**Fig 1Eii**). Increased fibrin deposition, another characteristic of glomerular endotheliosis, was also observed in kidneys from CBA/J x DBA/2 matings (**Fig 1Diii**) compared to CBA/J x BALB/c mice (**Fig 1Eiii**). Jones methenamine silver staining showed diffusely and irregularly thickened glomerular basement membranes in some glomeruli in CBA/J x DBA/2 mice (20%) (**Fig 1Div**) compared to BALB/c mated CBA/2 females (**Fig 1Eiv**). These renal lesions characteristic of human PE were only observed in the first pregnancy of DBA/2 mated CBA/J mice (data not shown).

### Blood pressure in DBA/2 mated CBA/J females

Hypertension is the most common diagnostic signs in PE and results from high peripheral resistance. However, no difference in mean arterial blood pressure (MAP) was observed in CBA/J x DBA/2 mice when compared to control matings (**Fig 2A**). MAP was slightly higher in the DBA/2 mated CBA/J females from day 8 to day 15 of pregnancy but did not reach statistical significance when compared to control matings (**Fig 2A**). In women, blood pressure decreases during a normal pregnancy because of the decrease in peripheral vascular resistance. This pattern was also observed in control pregnant mice (CBA/J x BALB/c), where the mean change in MAP from mating day decreased between days 8 and 15 in BALB/c mated CBA/J mice. Although not statistically significant this diminution in MAP was not observed in CBA/J x DBA/2 mice (**Fig 2A**).

### Increased sensitivity to Ang II in CBA/J x DBA/2 mice

An increased sensitivity of the arteries to vasoconstrictor agents like angiotensin II (Ang II) has been described in PE. Thus, we investigated if increased hypersensitivity to Ang II was present in the CBA/J x DBA/2 model. In response to a bolus injection of Ang II, MAP increased by 32±5 mmHg in CBA/J x DBA/2 mice and 16±4 mm Hg in CBA/J x BALB/c control matings (**Fig 2A**). The increase in MAP in response to Ang II was statistically greater in CBA/J x DBA/2 mice compared to CBA/J x BALB/c mice (P<0.001). This pronounced and sustained pressor hypersensitivity to Ang II was observed in CBA/J x DBA/2 mice from days 12 to 18 of pregnancy. In addition, aortic rings from CBA/J x DBA/2 mice showed increased contractile response to Ang II (100 mmol/L) when compared to aortic rings from control CBA/J x BALB/c matings. Aortic rings from CBA/J x DBA/2 mice showed a 45±6% reduction in diameter compared to 22±4% in control matings (**Fig 2B**). Increased sensitivity to AngII was only observed in first CBA/J x DBA/2 pregnancies. Second and third pregnancies show a response to Ang II not different from control matings CBA/J x BALB/c (18%±4 diminution in diameter).

### Effect of wheel running on blood pressure

In the previous set of experiments MAP was monitored in CBA/J x DBA/2 and CBA/J x BALB/c at rest. In this set of experiments, day 12 pregnant mice were subjected to wheel running for a period of 5 minutes. MAP was recorded before and after wheel running. Wheel running induced a MAP increased only in preeclamptic CBA/J x DBA mice (15±3 mmHg) when compared to control matings (2±1 mmHg). CBA/J x DBA/mice did not show hypertension during pregnancy. However, stimulus like Ang II or exercise triggered hypertension in these mice.

### Leptin levels in CBA/J x DBA/2 mice

To substantiate the characterization of our mouse model of PE we measured plasma leptin levels. Plasma leptin levels are significantly elevated in pregnant women with PE and the increase in plasma leptin levels is correlated with the severity of preeclampsia [14]. Plasma leptin levels increased through pregnancy in both CBA/J x DBA/2 and control matings but in CBA/JxDBA/2 mice a bigger increase was observed at day 12 of pregnancy (**Fig 2C**). This increase in plasma leptin levels observed in CBA/J x DBA/2 matings was maintained until delivery. After delivery the leptin values dropped to non pregnant levels (NP) in CBA/J x DBA/2 and control CBA/J x BALB/c matings (**Fig 2C**).

### Pravastatin prevents preeclamptic features in CBA/J x DBA/2 mice

**Figure 3:**
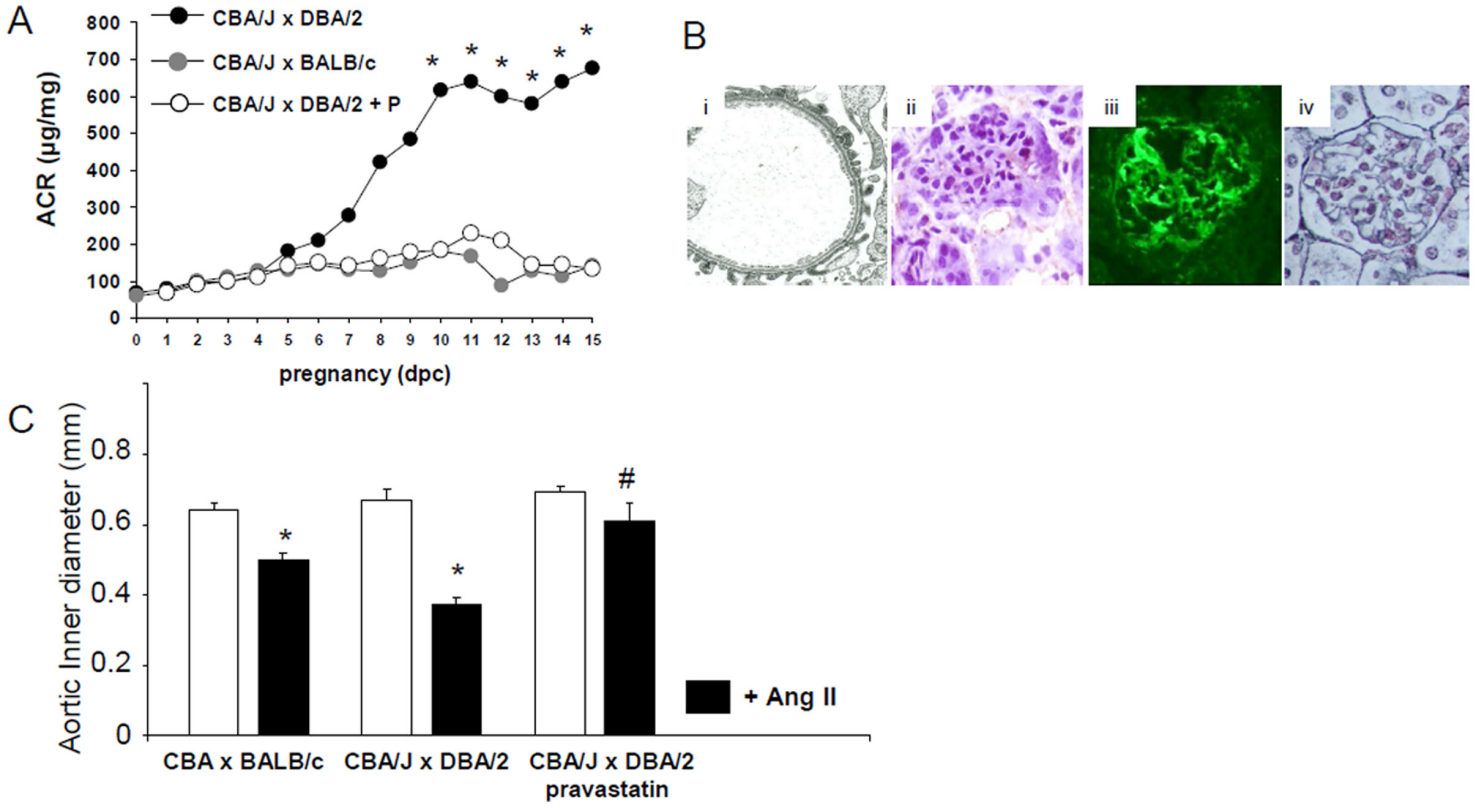
Pravastatin prevents glomerular injury and hypersensitivity to angiotensin II in CBA/J x DBA/2 mice. (A) Increased ACR levels were observed in CBA/J x DBA/2 mice along pregnancy (* p<0.01). Treatment with pravastatin abrogated albuminuria in CBA/J x DBA/2 mice (B) CBA/J x DBA/2 mice treated with pravastatin showed no signs of renal endothelial damage. (Bi) EM studies (original magnification 10,000 x) showed well preserved endothelial cells and open capillaries lumen in CBA/J x DBA/2 mice treated with pravastatin. (Bii) Increased fibrin deposition was not observed in glomerular capillaries from DBA/2 mated CBA/J mice that received pravastatin. (Biii) Pravastatin restored renal blood flow in CBA/J x DBA/2 mice. (Biv) Jones methenamine silver staining shows no signs of endotheliosis in CBA/J x DBA/2 mice treated with pravastatin. (C) Aortic rings from CBA/J x DBA/2 mice treated with pravastatin did not show increased contractile response in response to Ang II when compared to untreated CBA/J x DBA/2 mice (*p<0.05). N = 5–8 mice/experimental group.

Knowing that pravastatin, by diminishing tissue factor levels, prevented miscarriages and growth restriction in the CBA/J x DBA/2 model [6], we sought to investigate whether pravastatin can prevent the onset of key features of PE in CBA/J x DBA/2 mice. In accord with our previous findings, we found that pravastatin attenuated glomerular injury in CBA/J x DBA/2 mice. **Figure 3A** shows that ACR levels in CBA/J x DBA/2 mice were reduced by pravastatin. Accordingly, EM analysis of kidneys from these mice revealed normal configuration of glomerulus (**Fig 3Bi**). The capillary lumina were not occluded and there were no signs of endothelial injury. The endothelium was thin and the fenestrations were abundant and well preserved (**Fig 3Bi**). Fibrin deposition and decreased glomerular blood flow were not observed in the CBA/J x DBA/2 mice that received pravastatin. (**Fig 3Bii and 3Biii**). Similar to the EM studies, Jones methenamine silver staining showed no abnormalities in the glomerular basement membranes (**Fig 3Biv**).

CBA/J x DBA/2 mice treated with pravastatin did not show hypertension in response to Ang II or wheel running when compared to untreated CBA/J x DBA/2 mice (response to Ang II: 12±3 vs 32±5 mmHg, p<0.01; wheel running: 2±2 vs 15±3 mmHg, p<0.05). Aortic rings from CBA/J xDBA/2 mice that were treated with pravastatin showed a contractile response to Ang II not different from control matings (**Fig 3C**).

### Pravastatin increased free plasma VEGF levels

**Figure 4:**
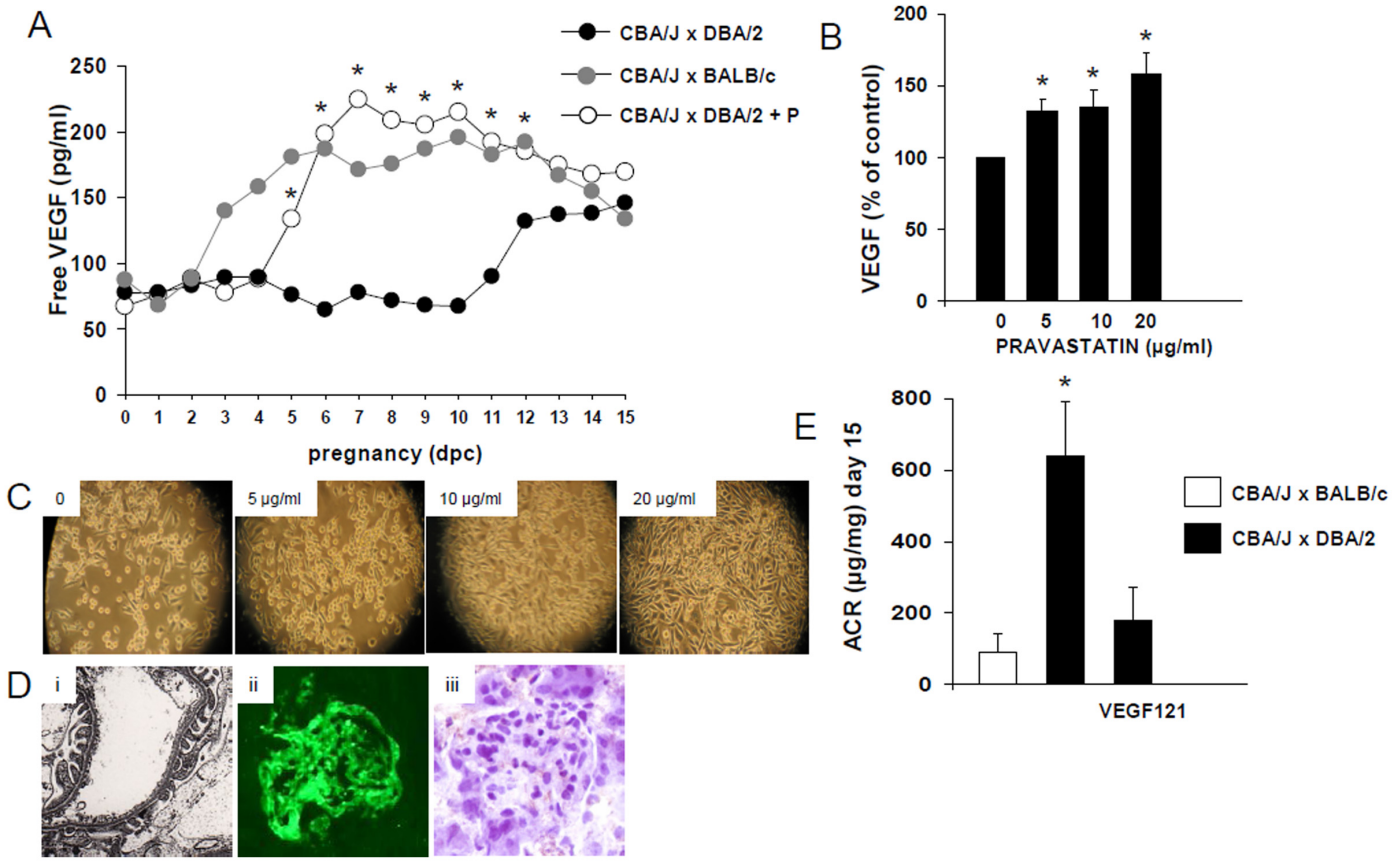
Pravastatin restores angiogenic balance in CBA/J x DBA/2 mice. (A) Pravastatin restored free plasma VEGF levels in CBA/J x DBA/2 mice (*p<0.05). (B) SM9-1 trophoblast cells incubated with pravastatin showed increase VEGF release in a dose dependent manner (*p<0.05). Values are expressed as percentage of control values.(C) Pravastatin (5- 10-20 µg/ml) increased cell proliferation in SM9-1 trophoblasts in a dose-dependent manner. (D) CBA/J x DBA/2 mice treated with VEGF 121 showed no signs of endotheliosis. (Di) electron microscopy studies show well preserved glomerular endothelial cells in CBA/J x DBA/2 mice treated with VEGF121.original magnification 10,000 x (Dii) Adequate blood perfusion comparable to CBA/J x BALB/c control matings was observed in CBA/J x DBA/2 mice treated with VEGF121. (Diii) Increased fibrin deposition was not observed in glomerular capillaries from DBA/2 mated CBA/J mice that received VEGF121. (E) Pravastatin ameliorated ACR in CBA/J x DBA/2 mice at day 15 of pregnancy (* p<0.01). N = 6–8 mice/experimental group.

We previously demonstrated a severe angiogenic imbalance in DBA/2 mated CBA/J mice, characterized by increased plasma sFlt-1 levels and low free VEGF levels [5]. CBA/J x DBA/2 mice that received pravastatin showed free plasma VEGF levels comparable to control matings (CBA/J x BALB/c) (**Fig 4A**). Previous results from our laboratory showed that pravastatin prevents sFlt-1 release from macrophages and thus rescue pregnancies [6]. Here we investigated if pravastatin can increase VEGF levels by stimulating VEGF release from trophoblasts. **Figure 4B** illustrates the effects of pravastatin (5, 10 and 20 µg/ml) on VEGF release by mouse trophoblasts SM-9. The basal trophoblast VEGF secretion was increased by pravastatin in a dose-dependent-manner. Pravastatin increased SM9-1 trophoblast proliferation in a dose-dependent manner (**Fig 4C**). Pravastatin restores VEGF levels in the CBA/J x DBA/2 model by a dual mechanism: inhibition of sFlt-1 release from macrophages [6] and stimulation of VEGF release from trophoblasts.

### VEGF121 reduced glomerular injury and abrogated albuminuria in CBA/J x DBA/2 mice

To confirm that pravastatin prevents PE in the CBA/J x DBA/2 mice by restoring the levels of VEGF, we treated DBA/J mated CBA/2 females with VEGF121. It has been reported that delivery of recombinant VEGF121 attenuates hypertension and prevents kidney damage in rat models of PE characterized by chronic elevations of sFlt-1 [15], [16]. Chronic administration of VEGF121 restored circulating VEGF levels to the levels observed in CBA/J x BALB/c mice with normal pregnancies (data not shown). Glomerular injury observed in CBA/J x DBA/2 mice was not observed in mice that received VEGF121 treatment (**Fig 4Di**). In addition, VEGF121 chronic treatment also restored glomerular blood flow and diminished fibrin deposition in glomerular capillaries in CBA/J x DBA/2 mice (**Fig 4Dii and iii**). The protective effects of VEGF121 on glomerular endothelium were also reflected in the absence of albuminuria. On day 15 of pregnancy, ACR levels in CBA/J x DBA/2 mice treated with VEGF121 were not different from values in control matings (**Fig 4E**). Increased hypersensitivity to Ang II vasoconstrictor effects was not observed in CBA/J x DBA/2 mice that received VEGF121 compared to CBA/J x DBA/2 untreated mice (MAP 10±3 vs 32±5 mmHg, p<0.01). Chronic infusion of VEGF also prevented IUGR in CBA/J x DBA/2 mice. Fetuses from CBA/J x DBA/2 mice treated with VEGF121 had similar body weights to fetuses from control CBA/J x BALB/c matings (data not shown).

Aortic rings from CBA/J xDBA/2 mice treated with VEGF121 did not show increased contractile response to Ang II when compared to untreated CBA/J x DBA/2 mice (aorta inner diameter (mm): 0.71±0.02 vs 0.37±0.03, p<0.01) The contractile response to Ang II in pravastatin-treated mice was not different from control matings (0.69±0.02 mm).

## Discussion

The preeclampsia/eclampsia syndrome has been recognized since ancient times. PE is a serious complication of pregnancy that increases maternal and perinatal morbidity and mortality and it still affects approximately 10% of pregnancies. Despite considerable research effort, very little is understood about its etiology and pathophysiology, which are complex and multifactorial. For many years it was arguable if an animal model can be of use in preeclampsia studies, as it was a human disease that was not observed spontaneously in other species. In this study we report several novel findings. Foremost we report a new mouse model were the relevant key features of preeclampsia appear spontaneously without genetic [17], [18] or surgical manipulation [19] or the administration of any compound to induce the condition [7], [20]. The CBA/J x DBA/2 model, that recapitulates the key preeclamptic symptoms, helped us identify mediators of PE and identify possible therapies. The fact that CBA/J x DBA/2 mice develop preeclampsia-like disease with all the accepted pathological changes (hypersensitivity to Ang II, proteinuria and renal glomeruloendotheliosis suggests that this mouse model is indeed an appropriate model of human PE. Using this model, we found that pravastatin restores the angiogenic balance by increasing VEGF release from trophoblasts and thus ameliorates preeclamptic features and rescues pregnancies in this model.

PE is documented to occur primarily in first pregnancies and rarely in subsequent pregnancies. Therefore, the concept of primigravidity is the epidemiological cornerstone of this disease [21]. In our mice studies we found that only first pregnancies in CBA/J females mated with DBA/2 males were complicated by PE while subsequent pregnancies were not associated with the disease. These studies share the concept of primigravidity with human preeclamptic pregnancies. It was also proposed that PE is a disease of new couples, especially after a short period of sexual cohabitation, creating and alternative primipaternity model [11], [21], our studies also follow the primipaternity model as second and third pregnancies with the same male resulted in normal pregnancies.

PE has been termed the “disease of theories”, reflecting the confusion that surrounds the causes and pathophysiology of PE [22]. Because PE only occurs during pregnancy and its symptoms resolve after delivery, factors produced by the placenta are thought to initiate widespread inflammation, thrombosis and vasoconstriction [8], [23]. Maternal hypertension is one of the key features of PE in women. Mice show similar gestation stage-dependent changes in maternal blood pressure to human. During a normal pregnancy blood pressure actually falls during gestation in humans and mice as well [24], [25]. Wong and colleagues reported that arterial blood pressure was transiently reduced in midgestation (9–13 days) in control pregnant mice [25]. A similar pattern was observed in control CBA/J x BALB/c mice with normal pregnancies. Despite the similarities in blood pressure during gestation in mice and humans, we were unable to demonstrate blood pressure increase in the CBA/J x DBA/2 mice with abnormal pregnancies. We only observed a slight increase in blood pressure that did not reach statistical significance. The absence of hypertension in this model can be due to the method used to monitor BP. However, the fact that the non invasive tail cuff technology was validated by comparison to simultaneous radiotelemetry blood pressure measurements [26] makes this unlikely. We also need to consider that we did not monitor blood pressure continuously; MAP was recorded for a short period of time each day. Another possible reason to explain the absence of hypertension in CBA/J x DBA/2 mice when compared to women is the different gestation time. Gestation in mice lasts 20 days, with formation of the beating heart and vascularized placenta completed by day 10. Therefore, the events of later development that take the last 8 months of gestation in humans occur in only the last 9 days in mice. Thus, there is a rather short time for hypertension to develop in mice. As such, it is possible that hypertension observed in human disease may not occur in mice due to the short time periods. Because there are descriptions of clinical presentations with pregnancy-induced features of PE but without hypertension in women [27], [28], the absence of hypertension does not reduce the usefulness of the CBA/J x DBA/2 model because it is clearly a pregnancy induced disorder with strong preeclamptic features.

Even in the absence of hypertension, we demonstrated an increased vascular susceptibility to vasoconstriction. CBA/J x DBA/2 mice showed increased sensitivity to Ang II and exercise when compared to control CBA/J x BALB/c matings. The increased response to Ang II observed in CBA/J x DBA/2 mice can be also caused by decreased formation of vasodilators such as nitric oxide (NO). We previously demonstrated a marked diminution in plasma NO levels during pregnancy in DBA/J mated CBA/J females when compared to control matings [6]. These data are consistent with the observations in woman, as enhanced vascular sensitivity to an angiotensin infusion can identify women who are at increased risk for the development of PE [29], [30]. An heterodimer of the G-protein−coupled angiotensin II type I and bradykinin B_2_ receptors might be involved in the molecular mechanism of this phenomenon [31]. Zhou and collaborators have shown that women with PE possess autoantibodies, termed AT(1)-AAs, that bind and activate the angiotensin II receptor type 1a [32]. In addition, they showed that key features of PE appeared in pregnant mice after injection with purified AT(1) AAs from women with PE [32].

In PE, there is a reduced invasion of trophoblasts and an incomplete transformation of the maternal spiral arteries [9], [10] causing the release of factor(s) from the defective placenta into the maternal circulation [23]. These factors are believed to induce the endothelial dysfunction underlying the main characteristics of preeclampsia [7]. sFlt-1, the soluble receptor for VEGF, seems to play an important role in the onset of maternal vascular disease in PE [7], [8]. We previously showed the crucial role of sFlt-1 and angiogenic imbalance in the CBA/J x DBA/2 model [5], [6]. Endotheliosis is a frequent renal lesion observed in patients with PE. Morphometric analysis of the kidneys of CBA/J x DBA/2 mice revealed significant ultrastructural differences when compared to control mating, including swelling of the glomerular endothelial cells, fibrin deposition and occlusion of capillary lumens. These renal features are consistent with endotheliosis. Moreover, reduced renal blood flow, also present in women with PE, was also observed in the CBA/J x DBA/2 mouse model of PE.

The presence of proteinuria confirms the diagnosis of PE and the amount of protein excreted in the urine is related to the severity of the disease in women. In CBA/J x DBA/2 mice we observed a time course increase of albumin to creatinine ratio, reaching maximum values at day 15 of gestation. Proteinuria in preeclamptic women usually develops at the end of the second trimester, beginning of the third trimester. Considering that the length of pregnancy in mice is 19-21 days, proteinuria in CBA/J x DBA/2 mice was observed at the same time period than in humans.

During pregnancy, leptin concentrations in the maternal circulation are elevated in both humans and rodents but decrease to pre-pregnancy levels at birth, suggesting a role for leptin in the maintenance of pregnancy [14]. Synthesis of leptin by the human placenta is established but whether the murine placenta synthesizes leptin remains controversial. We observed increased serum leptin levels in preeclamptic CBA/J x DBA/2 mice. However we could not identify the source of the increased leptin levels. Mouse trophoblasts incubates with leptin (data not shown) showed normal proliferation. The fact that leptin did not affect mouse trophoblasts growth in cell culture suggests that the increased levels of leptin observed in our PE model is rather a compensatory mechanism to maintain trophoblasts growth than the cause of trophoblast defective invasiveness.

A concept that preeclampsia may represent and exaggerated hyperinflammatory state that leads to destruction of trophoblasts was described more than 10 years ago by Redman and collaborators [10]. Haeger and colleagues suggest that the endothelial dysfunction in PE is caused by intravascular inflammatory reactions involving intravascular leukocytes as well as the clotting and complement systems [33]. Our observations are in agreement with these studies as we described an important role for inflammation (in particular complement activation) and thrombosis in the bad pregnancy outcomes in the CBA/J x DBA/2 model [5], [6]. We demonstrated that complement activation induces tissue factor expression on macrophages stimulating sFlt-1 release and causing placental angiogenesis failure and abnormal pregnancies.

Knowing that statins have anti-inflammatory effects [34] and protected pregnancies in the CBA/J x DBA/2 matings [6], we sought to investigate if statins can prevent the onset of PE-specific features in CBA/J x DBA/2 mice. Treatment with pravastatin prevented the appearance of the key features of PE in CBA/J x DBA/2 mice. Glomerular endothelial lesions and proteinuria were not observed in CBA/J x DBA/2 mice that were treated with pravastatin. That treatment with pravastatin during early stages of pregnancy (from day 4 to 12) prevents the onset of the maternal vascular disease of PE observed later in pregnancy suggests that poor placental development may be the underlying cause of PE. We previously described diminished giant trophoblasts cells in CBA/J x DBA/2 mice which suggests a deficient trophoblast invasion during placentation [5].

CBA/J x DBA/2 mice treated with pravastatin did not show increased susceptibility to Ang II pressor effects. In addition the contractile response of aortic rings to Ang II in CBA/J x DBA/2 mice treated with pravastatin was not different from control matings. This is in agreement with studies suggesting that statins lower blood pressure in hypertensive patients [35].

In vitro studies emphasize the beneficial effects of pravastatin in the treatment of preeclampsia. Increased VEGF release was observed in mouse trophoblasts incubated with increasing doses of pravastatin. We previously demonstrated that pravastatin inhibited the release of sFlt-1 from macrophages [6]. Thus pravastatin may prevent PE-specific features by restoring angiogenesis and placental development through two different mechanisms: decreasing sFlt-1 levels and increasing VEGF levels. Other authors also demonstrated that statins decreased the release of sFlt-1 from endothelial cells and normal –term placental villous explants [36].

In conclusion, we described a new mouse model of PE, where the relevant key features of human preeclampsia appear spontaneously. The CBA/J x DBA/2 model, that recapitulates this complex disorder, helped us identify pravastatin as a candidate therapy to prevent preeclampsia and its related complications. Clinical trials are needed to confirm its application to humans.

## Materials and Methods

### Mice matings and treatment protocol

Inbred CBA/J (H-2k), DBA/2 (H-2d), and BALB/c (H-2d) mice from The Jackson Laboratory (Bar Harbor, ME) were used in all experiments. Eight- to 10-week-old virgin female CBA/J mice were mated with 8- to 14-week-old BALB/c or DBA/2 males. Females were inspected daily for vaginal plugs; sighting a vaginal plug was designated as day 0 of pregnancy.

A group of CBA/J x DBA/2 and CBA/J x BALB/c mice were treated with pravastatin (20 ug/kg, sc) daily from day 4 to 12 of pregnancy. Another group of mice received vascular endothelial growth factor (VEGF) 121, the most soluble of VEGF isoforms, subcutaneously through a mini osmotic pump (Alzet, Cupertino, CA) (20 µg/kg/day) from day 4 to day 10 of pregnancy. Minipumps filled with VEGF121 in sterile PBS were implanted on day 4 of pregnancy under isofluorane anesthesia. Blood and urine samples were collected at predetermined intervals (from day 0 until delivery). Plasma leptin levels were measured by ELISA (Cayman Chemical Company, Ann Arbor, MI). VEGF plasma levels were determined by ELISA (R&D Systems Inc, Minneapolis, MN).

On day 15 a group of mice were sacrificed and aortic rings were isolated. Kidneys were also harvested to investigate the presence of endotheliosis. The frequency of fetal resorption and fetal weight were also determined on day 15 as previously described [5], [6].

To study the effect of primiparity on the development of preeclampsia, a group of CBA/J x DBA/2 mice completed their first or second pregnancy and were mated again with the same male. Litter sizes were recorded immediately after birth. A group of these mice were sacrificed on day 15 and fetal resorption frequency was calculated as previously described [5], [6]. Procedures that involved mice were approved by the York College – CUNY- Committee on Animal Use in Research and Education and were conducted in strict accordance with guidelines for the care and use of laboratory research animals promulgated by the NIH.

### Blood Pressure Measurements

Blood pressure was measured in the tail artery in pregnant CBA/J x BALB/c mice and CBA/J xDBA/2 mice with and without pravastatin or VEGF121 treatment at different times along pregnancy until postpartum. Measurements were performed using a computerized, non-invasive tail-cuff acquisition system (CODA System, Kent Scientific Corporation, Torrington, CT, USA) [26]. The CODA system utilizes volume-pressure recording technology to detect changes in tail volume that correspond to systolic and diastolic pressures and calculates mean arterial pressure (MAP) during each measurement cycle. Our protocol consisted of 8 acclimation cycles and 8 measurement cycles daily. Unanesthetized mice were placed in plastic holders. Mouse body temperatures was monitored closely and maintained between 34°C and 36°C using infrared heating. Readings differing by more than 10 mmHg were repeated after a rest period of 15-20 minutes.

A group of CBA/J x BALB/c and CBA/J x DBA/2 received a bolus injection of AngII (100 µL, 3 µmol/kg) via the retro-orbital vein. MAP was measured before and 10 minutes after Ang II injection. A group of mice was subjected to MAP monitoring before and after wheel running for 5 min.

### Assesment of Albumin/creatinine ratio (ACR)

Albumin-to-creatinine ratio (ACR) in random urine specimens (accepted alternative to 24-hour urine collections) was used to monitor kidney function. Urinary albumin was determined by ELISA (Albuwell M (Exocell, Philadelphia, PA). Creatinine in urine was quantified with the Creatinine Companion kit (Exocell, Philadelphia, PA), based upon the Jaffe' reaction of alkaline picrate with creatinine.

### Immunohistochemistry

Kidneys from every experimental group were acquired at day 15 of gestation and frozen quickly in O.C.T. compound (Sakura Finetek, CA). 10 µm-thick sections were cut and endogenous peroxidase activity was quenched with Peroxo-block (Invitrogen Corporation, Camarillo, CA). Sections were incubated with normal rabbit serum to block nonspecific binding (Cappel, Aurora, OH), then incubated with rabbit anti-mouse fibrin ((Dako North America Inc.,Carpinteria, CA), followed by incubation with specific secondary IgG antibodies conjugated with HRP (Sigma Chemical, St Louis, MO). Bound IgG-HRP was detected with diaminobenzidine. Sections were counterstained with hematoxylin.

Renal blood perfusion was examined by injecting pregnant females with 100 µL of 25 mg/mL FITC-labeled dextran (MW 2000000; Sigma-Aldrich, St Louis, MO) via the retro-orbital vein, at day 15 of pregnancy. After 15 minutes, the mice were killed and the kidneys removed and flash frozen. Serial frozen sections were examined and photographed under a fluorescence microscope (Nikon 50i) attached to a DigiSight Color digital camera system (Nikon DSRi1).

A group of kidneys were fixed in 2% paraformaldehye/2% glutaraldehyde in 0.1 M phosphate buffer for electron microscopy (EM). Kidney sections were evaluated for the presence of endotheliosis (loss of fenestrations, endothelial swelling and detachment of endothelial cells from the glomerular basement membrane). Jones methenamine silver staining was also performed in kidneys to identify glomerular lesions.

### Trophoblast cells culture

SM9-1 trophoblast cells, derived from a gestational day 9 Swiss -Webster mouse placenta [37], were cultured in RPMI-1640 supplemented with 2 mM glutamine, 1 mM sodium pyruvate, 2-mercaptoethanol (2-ME), penicillin/streptomycin and 10% FBS. Adherent SM9-1 trophoblasts cells were incubated with different concentrations of pravastatin (Sigma Chemicals, St Louis, MO) (5, 10 and 20 µg/ml). After 24 hours, cell proliferation was evaluated by light microscopy and VEGF levels were measured in the supernatants by ELISA (R&D Systems Inc, Minneapolis, MN).

### Isolation of aortic rings

CBA/J x BALB/c and CBA/J x DBA/2 mice untreated or treated with pravastatin were euthanized by cervical dislocation on day 15 of pregnancy. The abdominal-thoracic aorta was then excised, placed in cold PBS, and cleaned of adhering connective and adipose tissue. Aorta from each mouse was divided into 4 rings of 2-mm length. Each of the aortic rings was incubated in 2 mL DMEM medium containing either vehicle (dH2O) or Ang II (100 nmol/L) for 60 min. The inner diameters were calculated by measuring luminal diameter through the transverse section of the thin-vessel ring by using a calibrated micrometer eyepiece. Luminal diameters were measured at three points separated by equal angles and averaged. 5 mice were studied in each experimental group.

### Statistical analysis

Data are expressed as mean plus or minus standard deviation. After confirming that the data were normally distributed (Kolomogorov-Smirnov test of normalcy), statistical analyses were conducted using Student t test to compare differences in means. Associations were considered to be statistically significant if the value of P was less than 0.05. Data were processed using SigmaStat, version 3.1 (Systat, Point Richmond, CA), statistical program forWindows.
